# Pulsed Electromagnetic Fields in the treatment of fresh scaphoid fractures. A multicenter, prospective, double blind, placebo controlled, randomized trial

**DOI:** 10.1186/1471-2474-12-90

**Published:** 2011-05-06

**Authors:** Pascal Hannemann, Kevin WA Göttgens, Bob J van Wely, Karel A Kolkman, Andries J Werre, Martijn Poeze, Peter RG Brink

**Affiliations:** 1 Department of surgery, Maastricht University Medical Center, PO box 5800, 6226 AZ, Maastricht, The Netherlands; 2 Department of surgery, Radboud University Nijmegen Medical Center, Geert Groteplein zuid 10, 6525 GA, Nijmegen, The Netherlands; 3 Department of surgery, Rijnstate Hospital, Alysis Group, Wagnerlaan55, 6815 AD, Arnhem, The Netherlands; 4 Department of surgery, Canisius Wilhelmina Hospital, Weg door Jonkerbos 100, 6532 SZ, Nijmegen, The Netherlands

## Abstract

**Background:**

The scaphoid bone is the most commonly fractured of the carpal bones. In the Netherlands 90% of all carpal fractures is a fracture of the scaphoid bone. The scaphoid has an essential role in functionality of the wrist, acting as a pivot. Complications in healing can result in poor functional outcome. The scaphoid fracture is a troublesome fracture and failure of treatment can result in avascular necrosis (up to 40%), non-union (5-21%) and early osteo-arthritis (up to 32%) which may seriously impair wrist function. Impaired consolidation of scaphoid fractures results in longer immobilization and more days lost at work with significant psychosocial and financial consequences.

Initially Pulsed Electromagnetic Fields was used in the treatment of tibial pseudoarthrosis and non-union. More recently there is evidence that physical forces can also be used in the treatment of fresh fractures, showing accelerated healing by 30% and 71% reduction in nonunion within 12 weeks after initiation of therapy. Until now no double blind randomized, placebo controlled trial has been conducted to investigate the effect of this treatment on the healing of fresh fractures of the scaphoid.

**Methods/Design:**

This is a multi center, prospective, double blind, placebo controlled, randomized trial. Study population consists of all patients with unilateral acute scaphoid fracture. Pregnant women, patients having a life supporting implanted electronic device, patients with additional fractures of wrist, carpal or metacarpal bones and pre-existing impairment in wrist function are excluded. The scaphoid fracture is diagnosed by a combination of physical and radiographic examination (CT-scanning).

Proven scaphoid fractures are treated with cast immobilization and a small Pulsed Electromagnetic Fields bone growth stimulating device placed on the cast. Half of the devices will be disabled at random in the factory.

Study parameters are clinical consolidation, radiological consolidation evaluated by CT-scanning, functional status of the wrist, including assessment by means of the patient rated wrist evaluation (PRWE) questionnaire and quality of life using SF-36 health survey questionnaire.

Primary endpoint is number of scaphoid unions at six weeks, secondary endpoints are time interval to clinical and radiological consolidation, number of non-unions, functional status at 52 weeks and non-adherence to the treatment protocol.

**Trial registration:**

Netherlands Trial Register (NTR): NTR2064

## Background

Fractures of the scaphoid, which is the most commonly fractured of the carpal bones, represent 2-6% of all fractures and typically occur in young, active patients aged 15 to 40[1]. In the Netherlands 90% of all carpal fractures is a fracture of the scaphoid bone. Exact incidence is unknown, but in the Netherlands 21.000 scaphoid fractures are suspected and subsequently treated as such each year[2,3]. Probably 15 to 20% represent real fractures[4].

The scaphoid bone articulates with 5 surrounding bones in the wrist and therefore has an essential role in functionality of the wrist, acting as a pivot. Treatment of scaphoid fractures can be very troublesome and failure can result in avascular necrosis (up to 40%), non-union of the fracture (up to 21%) and subsequently early osteo-arthritis (up to 32%)[1,5-7]. In dislocated fractures complication rates are even higher[8]. Part of the explanation for this high incidence of avascular necrosis can be found in the special blood supply of the scaphoid bone. Branches of the radial artery enter the os scaphoideus dorsally, thus supplying the bone from distal to proximal. Therefore fractures of the mid or distal third of the scaphoid can result in avascular necrosis of the proximal part of the bone. Non-union is defined as the absence of healing at four to six months after injury. This may be due to delay in treatment, inadequate immobilization, localization of the fracture, instability due to displacement of the fracture fragments or combination with ligamentous injury of the carpus. All these conditions are responsible for severe impairment in wrist function and even permanent disability. Studies have shown that untreated non-unions of the scaphoid will lead to osteo-arthritis in 75% of all cases within 6-9 years and even up to 100% in 10 years, usually leading to permanent disability[9]. Therefore, treatment failure may have severe socio-economical consequences.

In addition impaired consolidation of scaphoid fractures results in longer immobilization and loss of function. Since the patient population consists mainly of young, productive people, prolonged immobilization and function loss leads to more days of lost at work with again more economical and social consequences. Studies showed that even uncomplicated healing leads to a mean employment interruption of 155 days. In case of complicated healing conditions like non-union of the fracture, the median period of disability is even 296 days[10].

Current treatment strategies are unable to deal with this problem. The number of complications following conventional treatment, being immobilization in a cast is, as mentioned before, quite high and surgery is generally performed only if complications in healing occur. Results of operative treatment are variable [11] and furthermore operative treatment for complicated healing of scaphoid fractures (e.g. delayed or non-union) is often initiated in a late phase, most often months after the fracture occurred, which again can have severe socio-economical consequences[10].

The exact incidence of scaphoid fractures in the Netherlands is difficult to estimate. On the one hand, literature indicates that approximately 24.000 carpal fractures (of which 21.000 scaphoid fractures) are being suspected[1-3]. On the other hand, the estimated incidence of true scaphoid fractures is 35 per year in a hospital with an adherence of 250.000 people. Furthermore, literature suggests that true incidence among suspected fractures of the scaphoid is around 15 to 20%[4,12,13]. On a national level, this would indicate that the total number of diagnosed scaphoid fractures amounts to approximately 3.000. Previous studies showed that even uncomplicated healing leads to a mean employment interruption of 155 days or 22 weeks[10]. The hypothesis is that the use of PEMF can shorten this duration by 30 percent[14]. If we assume that 25% of all scaphoid fractures could be treated with PEMF, then this would lead to an increase in total treatment costs of 750 (25% of 3.000) * €1.250 (costs of PEMF) = €937.500.

Still, from a societal perspective, the use of PEMF could potentially lead to a decrease in workdays lost of 750 (25% of 3.000)* 46 days (30% of 155 days) = 34.500 days. If we calculate the costs of these workdays lost by using the friction cost method, then this reduction could potentially lead to cost-savings of 34.500 * €303.21(cost per day of work lost based on the average of the productivity costs per hour as reported in Oostenbrink et al [15] assuming a work day of 8 hours) * 0.8 (correction factor) = €8.368.596

Physical forces used in fracture healing are direct current, Pulsed Electromagnetic Fields (PEMF) and ultrasound. An important hypothesis in the application of physical forces on fracture sites is that strain-generated electrical potentials may be a regulatory signal for cellular processes of bone formation[16]. Inductively-coupled electromagnetic fields have been used in medicine since 1974[17]. Several studies showed that physical forces stimulate osteogenesis, in that callus was formed around the cathode[5,18]. The first double-blind study of application of PEMF was on fractures with delayed union of the tibia, which showed a significantly better healing rate than the control group[19]. In non-union scaphoid fractures Bora et al reported a 71% reduction in non-union within twelve weeks after initiating the electrical stimulation[20]. Later this rather invasive method was replaced by PEMF, a non-invasive technique.

Several clinical trials have been conducted to test whether physical forces can also be used in treating fresh fractures[21,22]. The use of pulsed electromagnetic fields (PEMF) in the treatment of ununited tibial fractures is a promising non-invasive technique which may offer an effective alternative to surgery[23]. However, its effect has never been investigated in fresh scaphoid fractures, although there is evidence that treatment of fresh scaphoid fractures by physical forces (ultra sound) accelerates healing by 30%[14].

We therefore want to investigate whether the use of PEMF in fresh scaphoid fractures accelerates consolidation and reduces the incidence of disabling wrist conditions like scaphoid non-union or osteonecrosis. When considering the patient group, influence of sex, age or cultural background on healing of scaphoid fractures has never been reported. Relating to patient compliance, a non-published pilot study using the PEMF device showed good patient compliance with a drop out percentage of less than 10%.

This study is the first double blind randomized, placebo controlled trial to investigate the effect of pulsed electromagnetic fields on the healing of fresh fractures of the scaphoid and investigate the effects of this treatment on consolidation and complications of treatment.

The aim of our study is therefore:

1. To determine whether the use of bone-growth stimulation by means of pulsed electromagnetic fields in acute scaphoid fractures will accelerate healing both clinically and radiologically.

Consequences of accelerated consolidation on time off work and social well-being will also be investigated.

2. To determine whether the use of PEMF in acute scaphoid fractures will decrease the incidence of non-unions and avascular necrosis of the scaphoid and therefore the number of secondary surgical interventions. The association of mal-union and non-union of the scaphoid fracture with clinical outcome will also be investigated.

3. To investigate the effect of PEMF in acute scaphoid fractures on functional outcome

4. To investigate the potential cost-effectiveness of PEMF from a societal perspective when compared with care as usual.

## Methods/Design

This is a multi center, double blind, randomized, placebo-controlled trial. Four centers will participate in this trial, investigating the effect of pulsed electromagnetic fields on union of fresh scaphoid fractures.

The estimated effect of the investigated intervention is as described previously. All unilateral scaphoid fractures, types A1, A2, B1, B2 and B3 (all stable and unstable acute fractures except the dislocated and comminuted ones according to the Herbert classification) will be included [24].

Exclusion criteria are pregnancy, presence of a life-supporting implanted electronical device, additional fractures of wrist, carpal or metacarpal bones and pre-existing impairment in wrist function. If necessary, pregnancy will be excluded by means of testing. Study parameters are radiological consolidation, clinical consolidation, quality of life, functional outcome and non-union of the fracture. Primary endpoint is radiological proof of union of the scaphoid fracture at six weeks after initiation of the electromagnetic stimulation. Secondary endpoints are radiological and clinical consolidation at 24 weeks (non-union), 52 weeks and functional outcome and quality of life at 52 weeks. Furthermore adherence to the treatment protocol will be checked. The choice for the primary endpoint is based on previous data from studies from Mayr [14], Heckman [21] and Kristiansen [22] where significant acceleration of healing in acute (scaphoid) fractures was based on data collected at six weeks after initiation of stimulation.

Trabecular bridging (CT sign confirming fracture healing) at six weeks showed 81% fracture healing in the group with stimulated fractures versus 55% in the control group[14].

If a power analysis is conducted on base of this information, a group of about 54 patients in each group is needed. (Type alpha error 0.05, type beta error 0.20, outcome intervention group 81%, outcome control group 55%, calculated drop out 15%). Taking the estimated incidence into account, a total study group of 100 to 110 patients should be included within a period of 12-24 months, among 4 medical centers with an adherence of about 1.5 million people. Secondary endpoints are important because long-term consequences of delayed or complicated healing can be reliably predicted at 52 weeks.

All patients suspected of having an acute scaphoid fracture will be treated with cast immobilization. Presence of a scaphoid fracture is diagnosed by a combination of physical and radiographic examination. Previous studies showed that conventional radiographic examination is inadequate in diagnosing a possible occult scaphoid fracture. (Immediately after injury, up to 65% of scaphoid fractures remain radiographically occult with conventional radiographic examination.)[25,26] Therefore, if no apparent fracture line is seen on the initial X-rays, a CT scan will be performed within 3 to 8 days to confirm the diagnosis. Criteria for a bone fracture on (multidetector) CT images are the presence of a sharp lucent line within the trabecular bone pattern, a break in the continuity of the cortex, a sharp step in the cortex, or a dislocation of bone fragments. Multidetector CT has an unusually high acuratesse in detecting scaphoid fractures, resulting in up to 95% sensitivity and 100% specificity for the identification of cortical involvement of scaphoid fractures [26,27]. Herbert's classification of scaphoid fractures will be used[28]. The criteria mentioned earlier will determine in- or exclusion from the trial.

The small PEMF device (supplied by commercial support) will be placed on the cast within five days after diagnosing the fracture and will be applied for 24 hours a day continuously. Dependent on fracture consolidation, the device will be removed after six to twelve weeks. The cast will be a lower arm cast with the first metacarpal bone and both phalanges immobilized. Since the position of the thumb and the hand have no adverse effect on the displacement of the fracture or it's consolidation, this neutral plaster is chosen [29,30].

Half of the PEMF devices will be disabled at random in the factory. These disabled devices will give outward signs of normal function but will not generate a signal. The investigators will be unaware of the device's functionality. The patients will not be able to determine whether the device is working or not. At study completion, device serial numbers will be used to determine which patients received a working device. The company supplying the PEMF-devices will have no knowledge of patient outcome. Follow up will take place at six, nine, twelve, twenty-four and fifty-two weeks after diagnosis of the fractured scaphoid. At these times the cast will be removed and physical and radiological examination will be performed to determine fracture consolidation. Physical examination includes investigating 4 separate items. The first item is presence of pain on local pressure on the anatomic snuffbox (ASB). The ASB is defined as the groove between the tendons of extensor pollicis longus on the ulnar side and extensor pollicis brevis and abductor pollicis longus on the radial side. Tenderness is elicited by digital pressure in the floor of this groove. Sensitivity of this test is 100%, yet its specificity is low (9%)[31]. Some studies however, report a specificity of up to 57% when testing specifically for tubercle tenderness[32]. The second item to be tested is tenderness with longitudinal compression of the scaphoid; tenderness is elicited by clasping the extended, mid-abducted thumb between the examiner's thumb and index finger and pressing towards the scaphoid. In some studies a specificity up to 80% is reported for this test[33].

The third item to be tested is wrist movement. Wrist movement is defined as the range of motion the wrist can make in dorsal flexion, palmar flexion, radial and ulnar deviation. These movements are recorded in degrees. The sum of these degrees is expressed as a percentage of that of the opposite wrist to take individual variation into account[34]. A loss of more than 25% of wrist movement is considered to be significant[35]. Last, grip strength is measured with help of a JAMAR dynamometer. Grip strength of both the injured and the non-injured site will be tested. All tests will be compared with the opposite unaffected side.

Since standard X-rays are unreliable in determining the degree of consolidation, additional CT-scans will be performed in the follow up after 6 weeks, 12 weeks, 24 weeks and 52 weeks. Quantification of fracture healing with the CT scan is based on formation of callus along the fracture and trabecular ridging[36]. The CT scan at 6 weeks will be important for determining the portion of fractures rapidly healed and determining outcome for the primary endpoint. The CT scan at 12 weeks is important for establishing the portion of patients with a scaphoid fracture showing signs of delayed (and eventually non-) union. The CT-scan at 52 weeks is particularly important for 2 reasons. First of all identification of delayed unions (longer than 4 months) can be confirmed. Secondly, if consolidation was established before, it can be checked at later follow up dates if that conclusion wasn't premature and the fractures are in fact healed. Former studies have pointed out that follow up may reveal non-unions whereas the fracture was considered to be consolidated before [37]. When the fracture has both clinically and radiologically consolidated the plaster will be removed. If the fracture has not consolidated; a new plaster will be made. The timing of the removal of the plaster can vary between six or twelve weeks. Only patients who need immobilization of the fracture (not consolidated fractures) will have a PEMF device on them, for in clinically and radiographically consolidated patients there is no need for further treatment. All patients will receive the same follow up, as described above. If the fracture is not consolidated after twelve weeks, at physical or radiographic examination, yet the patient has no pain, the treatment is finished. If the patient has got pain, he will get a removable splint.

In addition to the physical and radiographic examination, patients will be required to fill in a questionnaire: a SF health survey 36. The SF36 questionnaire appears to be a valid and reliable instrument to measure pain and psychosocial well-being[38]. This will be done after inclusion in the trial, before applying the PEMF device, at 6,9,12,24 and at 52 weeks. For assessment of functional deficit, disability and pain level, the Patient Rated Wrist Evaluation (PRWE) will be used. The PRWE is an easy 15-item questionnaire designed to measure wrist pain and disability in activities of daily living. The PRWE allows patients to rate their levels of wrist pain and disability.

Studies showed that the total PRWE's score's reliability was excellent over both the short term (2-7 days) and the long term (1 year)[39]. Patients will be asked to fill out this questionnaire at 6,9,12,24 and 52 weeks after inclusion.

**Figure 1:**
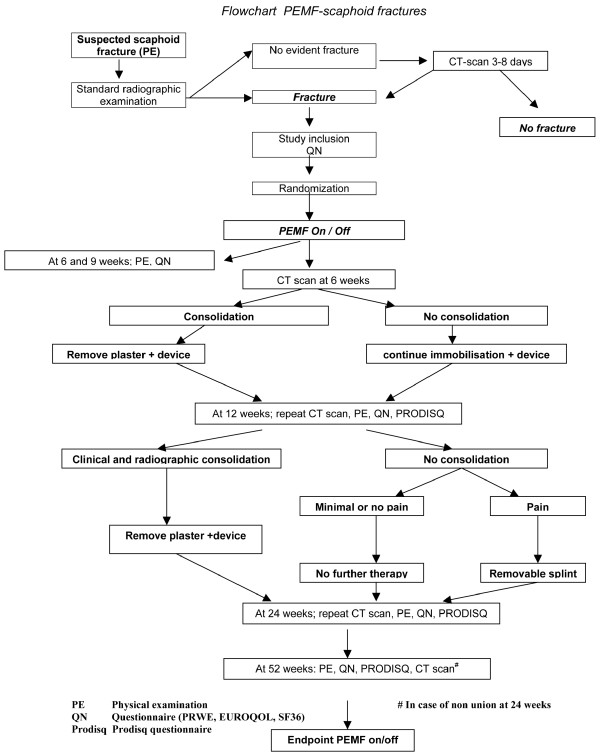
**Flowchart**.

Follow up will end 52 weeks after inclusion (Figure 1).

We have not conducted preliminary studies on this subject. Yet there is sufficient literature available proving the effectiveness of this intervention, as mentioned before. There is however some experience with the use of pulsed electromagnetic fields in the treatment of scaphoid fractures in the medical centers in Arnhem, Nijmegen and Hoorn. Considering the hospital in Hoorn, with an adherence of 175.000 patients, pulsed electromagnetic fields-treatment of 13 patients within a period of 9 months has taken place. In Nijmegen, where the participating hospital has an adherence of 275.000 patients, 37 patients with a scaphoid fracture were initially treated with PEMF within a period of 2 years. So concerning the quantitative feasibility of this study, if we extrapolate these numbers to the total region of 4 hospitals with an adherence of around 1.5 million patients, we suspect that inclusion of 100 patients within a period of 1 year should be possible.

Concerning the practical feasibility of the study, the device will be attached to the plaster with additional soft cast after the transducer coil has been applied on the skin above the fracture site. The patient cannot move or displace the device. Pilot studies using the PEMF device for the same indication have confirmed that most patients do not experience any inconvenience because of the device. No adverse effects of PEMF have been recorded. So no limitations are expected concerning the feasibility of the intervention.

The research question for the economic evaluation is:

Is the use of PEMF for fresh scaphoid fractures potentially cost-effective when compared with care as usual from a societal point of view?

The economic evaluation will include both a cost-effectiveness analysis and a cost-utility analysis from a societal perspective. For the cost-effectiveness analysis, the incremental cost-effectiveness ratio (ICER) will be calculated and expressed as the incremental costs per consolidation (see power-analysis). This means that the difference in total treatment costs between the intervention group and care as usual will be divided by their difference in effectiveness (i.e. consolidation). In addition, for the cost-utility analysis, the incremental costs per Quality Adjusted Life Years will be calculated. For this purpose, the health states of the SF36, a questionnaire developed to measure health related quality of life, will be converted to utilities.

### Regarding cost-analysis

Resource use of the following cost-categories will be measured:

Within health care: control visits, X-ray, CT-scan, emergency visits, costs of the intervention (i.e. device). Outside health care: productivity loss, travel costs. The total number of workdays lost will be measured by using modules of the PRODISQ, Productivity and Disease Questionnaire at baseline, 12, 24 and 52 weeks[40]. The costs of productivity loss will be calculated by means of the friction cost method, based on the average standardized wages per hour. The friction cost method takes into account the time, which is needed to replace a sick employee in order to restore the production at the original level [15]. Similar to the clinical study, the time horizon for the economic evaluation will be from the moment of inclusion until 12 months follow-up. Within the context of this study, no costs occur after one year, therefore discounting will not be applied. Total treatment costs will be calculated by multiplying volumes of use with the costs per unit. Unit costs will be derived from the hospital financial department or the Dutch guidelines for cost-calculation [15] Indirect costs (in this case general hospital overhead) will be allocated to the direct costs as an overall percentage, in accordance with the Dutch guidelines for cost calculation.

### Regarding the analysis

Data will be analyzed according to the intention-to-treat principle. Confidence intervals surrounding the mean differential costs will be calculated by the bootstrap method. This method estimates the sampling distribution of a statistic with replacement from original data [41] In addition, bootstrap analysis will also be used to quantify the uncertainty surrounding the incremental cost-effectiveness ratio (ICER) [42].

Results of this analysis will be presented in cost-effectiveness planes and acceptability curves. A cost-effectiveness plane is a graphical presentation of four situations or quadrants in which additional costs and additional health outcome effects of a new therapy are compared to care as usual. The acceptability curve shows the probability of a new therapy being more cost-effective than the usual treatments for various threshold values, i.e. the maximum amount society is willing to pay.

### Regarding patient outcome analysis

As described above, the primary outcome for the cost-effectiveness analysis will be the number of consolidated fractures. In addition, the outcome for the cost-utility analysis will be QALY, based on the scores of the SF-36. The SF-36 is developed to measure general health related quality of life [43]. The survey consists of 36 items and questions, which present respondents with choices about their perception of health over the last week. The following dimensions are included; physical functioning, role limitations due to physical problems, bodily pain, general health, vitality, social functioning, role limitations due to emotional problems and mental health. The scores from the SF-36 can be converted to utilities and hence to QALY's by using the scoring model as developed by Brazier J et al.[44]

### Ethics and consent

The study protocol conforms to the Helskinki Declaration and to local legislation. The local medical ethics committee [45] reviewed and approved the study protocol. All patients have to provide written informed consent before participation in the study.

## Competing interests

The authors declare that they have no competing interests.

## Authors' contributions

PH designed the present study protocol, and drafted and revised the manuscript, and is the main study coordinator. KWAG drafted and revised the manuscript for publication and participated in the statistical design of the study. BJW participated in designing the original study protocol and participates in the coordination of the study, and revised to manuscript. KAK participated in designing the original study protocol and participates in the coordination of the study, and revised the manuscript. AJW participated in designing the study and participates in the coordination of the study, and revised the manuscript. MP set up the statistical design of the study and revised the manuscript. PRGB revised the study protocol and manuscript. All authors read and approved the final manuscript.

## Pre-publication history

The pre-publication history for this paper can be accessed here:

http://www.biomedcentral.com/1471-2474/12/90/prepub
